# Reported use of evidence in clinical practice: a survey of rehabilitation practices in Norway

**DOI:** 10.1186/s12913-018-3193-8

**Published:** 2018-05-25

**Authors:** Jennifer L. Moore, Svein Friis, Ian D. Graham, Elisabeth Troøyen Gundersen, Jan E. Nordvik

**Affiliations:** 1 Regional Center of Knowledge Translation in Rehabilitation, Sunnaas Rehabilitation Hospital, Oslo/Nesodden, Norway; 2 Institute for Knowledge Translation, Carmel, IN USA; 3 Institute of Clinical Medicine, University of Oslo, Oslo, Norway; 4 University of Ottawa, Ottawa, ON Canada; 5 Ottawa Hospital Research Institute, Ottawa, ON Canada; 6 Unicare Fram, Rykkinn, Norway

**Keywords:** Evidence-based practice, Knowledge translation, Implementation, Rehabilitation, Outcome measures, Physical therapy, Occupational therapy, Speech language pathology

## Abstract

**Background:**

The South Eastern Health Region in Norway serves approximately 2.8 million people, which is more than half of Norway’s population. Physical medicine and rehabilitation services are provided by 9 public hospital trusts and 30 private rehabilitation facilities. The purposes of this study were to conduct a psychometric analysis of the EBP Implementation Scale (EBPIS) and describe rehabilitation clinicians’ self-reported 1) use of evidence-based practices (EBPs), 2) use of EBPs across hospitals, and 3) determine factors associated with use of EBPs in the South Eastern Health Region in Norway.

**Methods:**

A cross-sectional study using an online survey was conducted with public hospitals and private rehabilitation centers. The survey, which was distributed throughout the region, included the EBPIS, 8 questions related to EBP in the health region, and demographics. Response frequencies were calculated and described. Internal consistency and factor structure of the EBPIS and its subscales were determined. Associations and differences in groups with similar demographics, EBPIS scores, and use of EBPs were identified.

**Results:**

A total of 316 individuals completed the survey, including allied health clinicians, nurses, psychologists, social workers, and physicians. The EBPIS mean score was 30/72. A factor analysis identified that the EBPIS can be divided into 3 subscales: literature search and critical appraisal (α = .80), knowledge sharing (α = .83), and practice evaluation (α = .74). EBP activities reported were primarily related to literature searches, critical appraisal, and discussing evidence. Approximately 65 and 75% of respondents agreed that the same OMs and evidence based interventions were used within the local clinic respectively. Fewer agreed that the same OMs (13%) and evidence-based interventions (39%) are used regionally.

**Conclusion:**

The EBPIS and its subscales demonstrated excellent internal consistency. Practice variability exists in rehabilitation throughout Southeastern Norway. An increased emphasis on use of EBP throughout the region is needed.

## Background

Evidence-based practice (EBP) is the use of the best available evidence, clinical expertise and patient values to guide health care decisions [1]. Steps to perform EBP include the construction of a clinical question, a literature search, critical appraisal, application of evidence into practice, and evaluation of the outcome of the practice [1]. Literature on EBP has also emphasized the importance of administering an evidence-based treatment with fidelity, which indicates the degree to which treatment administration corresponds to the “prototype treatment” (i.e. protocol/treatment studied in the literature) [2]. Fidelity refers to appropriate use of measurements to assess patients and monitor progress as the result of an intervention, as well as the application of a treatment intervention in clinical practice [2]. While administering an EBP with high fidelity is important, the need to adapt a research-based intervention for clinical implementation has also been recognized [3]. While EBP is considered critical to high quality care, provision of health care that is based on experience instead of evidence has been observed across all disciplines in primary and specialty health care [4]. These practices may lead to health care that is less efficient [5], less effective [6], and may limit health outcomes [7].

Another related concept is Knowledge translation (KT), which is the “*a dynamic and iterative process that includes synthesis, dissemination, exchange and ethically-sound application of knowledge to improve the health of [the population], provide more effective health services and products and strengthen the health care system”* [8]. Importantly, KT includes the dissemination of knowledge and a process that promotes the use of EBP. Several studies have examined the use of EBP in physical medicine and rehabilitation. These studies indicate that 30 to 40% of patients did not receive care that was congruent with evidence, 20–25% of care was unnecessary or potentially harmful [9], and over 90% of clinicians chose treatments based on what they learned in school or read in textbooks [10–12]. In a survey of 244 physical therapists (PTs), respondents indicated they used evidence during decision-making 0 to 1 times (33.8%), 2 to 5 times (52.9%), and 6 or more times (13.3%) each month [13]. Use of EBP may be similar across practice settings and geographical locations [11], but could vary by level of independence performing tasks [14]. Clinician-related barriers to EBP include limited access to full-text articles, lack of understanding of how to critically appraise and apply evidence, and time limitations that prohibit finding and evaluating articles [15–17]. Facilitators of EBP include use of a multi-level approach that targets identified barriers [18], positive attitudes and motivation to use EBP [19], leadership support [19], and access to online EBP resources [20].

Even though EBP is valued, the translation of evidence into practice is a slow process [21, 22]. Use of theoretical frameworks, such as the Knowledge-to-Action (KTA) framework [18], may expedite KT. The KTA framework includes two major components, which are knowledge creation and the action cycle. Knowledge creation includes the development of primary research articles, synthesis of research, and creation of user-friendly documents. The action cycle is the process by which knowledge is implemented. The process includes problem identification, local adaptation of evidence, assessment of facilitators and barriers, KT interventions, monitoring use, outcomes assessment, and sustaining the new practice. Each phase includes a multi-level approach and incorporates various stakeholders’ perspectives. Implementing EBP also requires a systematic process that includes multiple stakeholders and an organizational infrastructure to support KT [23, 24]. As indicated by the KTA framework, the first step in KT is identification of the problem. Therefore, more knowledge is needed about routine use of EBPs by individuals, organizations and health systems who specialize in the field of physical medicine and rehabilitation, such as PTs, occupational therapists (OTs), speech language pathologists (SLPs), and nurses.

One method of assessing a healthcare provider’s use of EBP is through validated surveys such as the EBP Implementation Scale (EBPIS) [25, 26]. This self-report scale asks respondents to indicate the number of times components of EBP were performed over the past 8 weeks. Respondents rate frequency of performance on a 5 point scale, where 0 equals “0 times” and 4 indicates “> 8 times.” The total score, which could range from 0 to 72, is obtained by summing the responses on each question. A higher score indicates a greater frequency of use of EBP. Results from the EBPIS are often reported in three categories, such as critical appraisal of research studies, sharing evidence, and evaluation of one’s own practice [26]; however, the original study identified a unidimensional construct [25]. This should be examined further to confirm the constructs assessed by this scale.

The South Eastern Health Region in Norway serves approximately 2.8 million people, which is more than half of Norway’s population. Physical medicine and rehabilitation services are provided by 9 public hospital trusts and 30 private rehabilitation facilities. In Norway, physical, occupational and speech therapists are trained in undergraduate programs and are able to practice after achieving an undergraduate degree. However, support is often provided to clinicians in Norway who are interested in pursuing a Master’s Degree or Doctorate in Philosophy in a related field. Research findings indicate that educational level may be associated with perceptions of EBP, barriers to EBP, and use of EBP in clinical practice [27–31]. While research has identified associations between advanced education and improved use and fewer perceived barriers to EBP, the impact of education on EBP activities warrants further investigation.

The primary purposes of this study were to conduct a psychometric analysis of the EBPIS and to describe the perspective of rehabilitation clinicians who practice in South-Eastern Norway related to use of EBPs within and across hospitals, and to determine factors associated with use of EBPs.

## Methods

To understand the use of EBPs in South-Eastern Norway, we developed an online survey that included a Norwegian translation of the EBPIS that was initially published by Stokke and colleagues [26], questions related to organizational and regional implementation of EBPs, and participant demographics. We first determined internal consistency and performed a factor analysis on the EBPIS. In addition to the EBPIS, we developed 8 self-report questions related to EBPs within clinics and throughout the South Eastern health region in Norway. These questions were designed to identify whether EBPs are delivered in a similar manner between clinicians and rehabilitation clinics throughout the region. The responses to the questions were rated on a 5 point Likert Scale, with “Strongly Agree” and “Strongly Disagree” as anchors (Table 1).

**Table 1:** Survey Questions about EBP in the Health Region. Responses were rated on a 5 point Likert Scale, with “Strongly Disagree” and “Strongly Agree” as anchors

Regional Questions	
1. I believe that clinicians use the same standardized outcome measures throughout South-East Norway. 2. I believe that clinicians use the same standardized outcome measures in the hospital or clinic where I work. 3. I believe that evidence based practice provides the best treatment for patients 4. I believe that there are evidence-based interventions that are delivered in a standardized way (similar doses and methods) throughout South-East Norway. 5. I believe that there are evidence-based interventions that are delivered in a standardized way (similar doses and methods) in the hospital or clinic where I work. 6. There is an expectation to incorporate EBP at the hospital or clinical where I work. 7. I have access to experts in EBP in my clinic. 8. I have access to experts in EBP in the health region.	

Last, we asked several demographic questions to understand participant characteristics that may be associated with use of EBPs. Questions included age group, years of practice, degree, and specialization. These demographics were compared against the EBPIS total score and the underlying subscales.

The survey was administered online (Enalyzer; Copenhagen, Denmark) and remained open for 31 days. An email invitation was distributed to 579 individuals who signed up to for a distribution list established by the Regional Center for Knowledge Translation in Rehabilitation, which is the KT center that serves the public and private rehabilitation facilities in the South Eastern Norway Health region. Individuals who did not respond to the survey were sent two reminders. An open invitation was distributed in the KT Center’s newsletter. Study approval was provided by the Data Protection Official at Oslo University Hospital (#2015/4496). All participants provided informed consent as the first step of the survey.

## Data analysis

Data were analyzed using SPSS 22.0 (Windows). Response frequencies were calculated and described. Data sets with missing items were excluded from the analysis. To determine the internal consistency and factor structure of the EBPIS, we used Cronbach’s Alpha and a factor analysis. Once underlying factors and subscales were identified, Cronbach’s alpha was calculated for each subscale.

To identify differences between demographically similar groups and EBPIS and subscale scores, we conducted a one-way analysis of variance (ANOVA) and a Tukey Kramer post-hoc test. These statistics were also used to identify differences in perceptions of use of EBPs within and across sites with greater than 10 participants. Levene’s test was used to test homogeneity of variances. We conducted a Pearson product-moment correlation to determine associations between the demographics and use of EBP.

## Results

A total of 494 surveys were initiated and 316 participants completed the EBPIS and regional questions. Accounting for the number emails sent, the response rate was a maximum of 55%. However, we are unaware of the number of individuals who clicked the link to view the newsletter that contained the invitation or if individuals forwarded the email invitation to others to respond. Therefore, total number of individuals who viewed invitation is unknown. Participants from 7 public hospitals and 27 private rehabilitation centers responded (Table 2). Eight of the participating hospitals had > 10 participants, with a mean of 26.33 (SD = 11.40) respondents. The mean total score and standard deviation on the EBPIS was 30 ± 10.6 out of 72 points.

**Table 2:** Demographics of survey respondents

Characteristic	Response (*n*)
Sex	
Female	246
Male	70
Age group (years)	
20–29	29
30–39	67
40–49	92
50–59	86
60+	42
Profession	
Administration	14
Physical Therapy	65
Occupational Therapy	39
Speech Language Pathology	7
Nurse	43
Nursing assistant	3
Physician	29
Psychologist	14
Social worker	19
Quality Improvement	14
Lab technician	3
Management	39
Behavioral therapist	1
Other	26
Years of practice	
< 5	50
5–10	45
11–15	45
> 15	176
Degree	
Baccalaureate	237
Master’s	61
Doctorate	18
Specialist	
Yes	122
No	138
Not relevant	56
Setting	
Inpatient	212
Outpatient	53
Not applicable	51
Interdisciplinary Team	
Yes	261
No	55

### Psychometric analysis of the EPB implementation scale

During this study, we examined the internal consistency of the EBPIS and conducted a factor analysis. The internal consistency analysis resulted in a Cronbach’s alpha of .90. Next, we performed a factor analysis on the EBPIS to determine dimensionality (Table 3, with loadings of <.30 omitted). The factor loadings suggest three underlying factors or subscales (Table 4): Literature Search and Appraisal (five items), Knowledge Exchange (six items), and Practice Evaluation (five items). Two items were removed because of high loading on two subscales. Results demonstrated excellent internal consistency for each scale, where Cronbach’s Alpha was .80 for Literature Search and Appraisal, .83 for Knowledge Exchange, and .74 for Practice Evaluation. The scales were moderately correlated, ranging from .37 (between Practice Evaluation and Literature Search and Appraisal) to .59 (between Knowledge Exchange and Literature Search and Appraisal). The mean scores and standard deviation for each of the scales were: Literature Search and Appraisal scale 7.9 ± 3.6 (maximum of 20), Knowledge Exchange 9.2 ± 3.8 (maximum of 24), and Practice Evaluation 6.0 ± 2.9 (maximum of 20).

**Table 3:** Factor pattern matrix. Factors with loadings of <.30 have been omitted

	Knowledge Exchange	Literature Search & Appraisal	Practice Evaluation
EBP1	**.54**		
EBP2		**.79**	
EBP3		**.63**	.34
EBP4	.59	.55	
EBP5	.36		**.57**
EBP6	.50	.50	
EBP7			**.56**
EBP8	**.78**		
EBP9	**.65**		
EBP10	**.79**		
EBP11	.46	**.68**	
EBP12		**.75**	
EBP13		**.51**	.35
EBP14	**.53**		.41
EBP15			**.66**
EBP16			**.80**
EBP17			**.76**
EBP18	**.65**		

Items in bold were assigned to an index and retained for the subscale (i.e. Knowledge Exchange, Literature Search and Appraisal, and Practice Evaluation)

**Table 4:** EBP implementation scale subscales

Literature Search and Appraisal (α = 0.80)	
EBP2. Critically appraised evidence from a research study	
EBP3. Generated a PICO question that is relevant to my clinical practice	
EBP11. Read and critically appraised a clinical research study	
EBP12. Accessed a database of systematic reviews	
EBP13. Accessed a Guidelines Clearinghouse	
*Score range: 0 to 20	
Knowledge Exchange (α = 0.83)	
EBP1. Used evidence to change my clinical practice	
EBP8. Shared an EBP guideline with a colleague	
EBP9. Shared evidence from a research study with a patient or family member	
EBP10. Shared evidence from a research study with a multidisciplinary team member	
EBP14. Used an EBP guideline or systematic review to change clinical practice where I work	
EBP18. Promoted the use of EBP to my colleagues	
*Score range: 0 to 24	
Practice Evaluation (α = 0.74)	
EBP5. Collected data on a patient problem	
EBP7. Evaluated the outcomes of a practice change	
EBP15. Evaluated a care initiative by collecting patient outcome data	
EBP16. Shared the outcome data collected with colleagues	
EBP17. Changed practice based on patient outcome data	
*Score range: 0 to 20	
Items Excluded from Subscales	
EBP4. Informally discussed evidence from a research study with a colleague	
EBP6. Shared evidence from a study/studies in the form of a report or presentation to 2 colleagues	

### Use of evidence-based practices

**Fig. 1:**
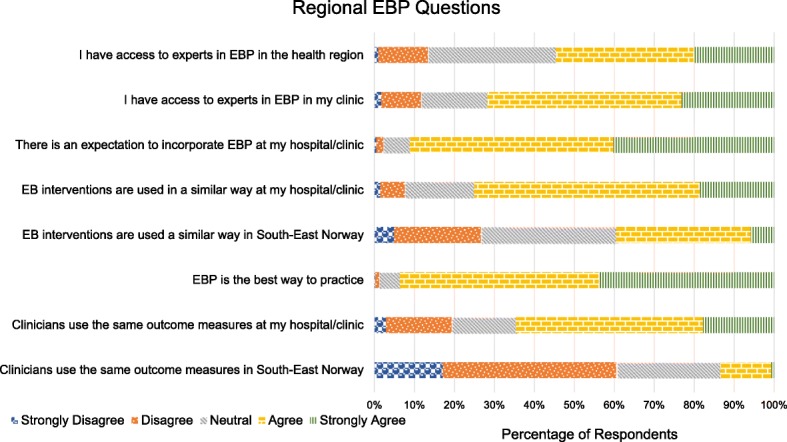
Regional EBP Questions

Figure 1 provides the survey responses related to use of EBPs within clinics and in the region. Approximately 65% agreed that clinicians use the same outcome measures (OMs) locally, but only 13% agreed that practice is throughout the region. Similarly, 75% agreed that clinicians administer evidence-based interventions in similar ways within a local site. However, only 39% agreed that the same evidence-based interventions are used within the region.

### Factors associated with use of evidence-based practices

A one-way ANOVA was conducted to identify differences in responses among each of the hospitals with > 10 participants on the regional questions. No significant differences (*p* > .05) were identified for 6 of the 8 questions. Significant differences were identified on 2 questions related to use of the same OMs locally (*p* < .001) and access to experts in the health region (*p* = .016). A post-hoc test revealed a higher number of respondents from one hospital reported “neutral” agreement (mean response = 3.0) when asked whether the same OMs are used within their hospital as compared to “agree” (mean response ~ 4.0); *p* < .037) at 5 other hospitals. Lastly, respondents from a large hospital reported significantly higher agreement (mean = 4, indicating agree) regarding access to experts in the health region than one other hospital (mean = 3.3, indicating neutral; *p* = .035).

To determine if the EBPIS total and subscale scores vary by demographics we conducted a one-way ANOVA. We examined the differences between degree and total score, in the following categories: bachelors, master’s, and doctorate. There was homogeneity of variances, as assessed by Levene’s test for equality of variances (*p* = .111). A significant difference was identified for individuals with different degrees and total score on the EBPIS (*p* < .0005) and the Literature Search and Appraisal (*p* < .0005) and Knowledge Exchange (*p* < .0005) subscales. The mean total score on the EBPIS increased from the bachelor degree (*n* = 236, 29.8 ± 9.9), to master’s degree (*n* = 61, 31.3 ± 8.4), to a doctorate degree (*n* = 18, 45.8 ± 14.2). Post-hoc testing revealed a statistically significant difference between the EBPIS total score and individuals with a bachelor and doctorate degree (*p* < .0005) and between individuals with a masters and doctorate degree (*p* < .0005). No significant differences were identified when comparing individuals with a bachelor and master’s degree (*p* = .543). Next, we examined data from participants with a bachelor’s degree to determine if differences in EBP may exist as a result of age or attainment of a specialty certification. The mean score on the EBPIS in individuals with a specialty certification was 31.7 points (SD = 1.1) as compared to 30.4 points (SD = .9) in individuals without specialty designation. A t-test identified significant differences on the EBPIS total score (*p* = .003), but no differences were identified on the subscales (*p* > .05).

A Pearson’s product-moment correlation assessed the relationship between the participant characteristics and EBPIS total and subscale scores. (Table 5) The relationship was linear with both variables normally distributed, as assessed by Shapiro-Wilk (*p* > .05). There was a small positive correlation between the total score and academic degree, *r* = .289, *p* < .0005, with degree explaining 8.1% of the variation in the score. Academic degree also demonstrated a small correlation with the Critical Appraisal (*r* = .379, *p* < .0005) and Practice Evaluation (*r* = .205, *p* < .0005) subscales. A small negative correlation was demonstrated between attainment of specialization and total score (*r* = −.121, *p* = .025) and the critical appraisal subscale (*r* = −.120, *p* = .026).

**Table 5:** Results of Pearson’s product-moment correlation analysis

Pearson Correlation for Study Variables					
	Specialists	Academic Degree	Work Experience	Sex	Age Group
EBP Implementation Scale	−.121*	.289*	.031	.092	−.013
EBP Critical Appraisal	−.120*	.379*	−.017	.061	−.044
EBP Knowledge Transfer	−.087	.205*	.027	.096	−.012
EBP Practice Evaluation	−.017	.055	.015	.001	−.024

Note: *p < .05

## Discussion

### Psychometric analysis of the EPB implementation scale

During a psychometric assessment of the EBPIS, we determined the scale’s internal consistency and identified three underlying subscales using a factor analysis. The results indicate that the EBPIS scale can be divided into 3 subscales that assess EBP steps, including Literature Search and Critical Appraisal (α = .80), Knowledge Sharing (α = .83), and Practice Evaluation (α = .74). The mean of each subscale was less than half of the maximum score (7.9/20 Literature Search and Appraisal; 9.2/24 Knowledge Exchange; and 6.0/20 Practice Evaluation), indicating that these EBP activities are performed less than 6 times over 8 weeks. While it may be reasonable to perform some activities at this frequency, such as those on the literature search and appraisal subscale, some activities on the other subscales should be a fundamental component of using EBP with patients. For example, collecting data (i.e. use of assessments and outcome measures), evaluating a care initiative by collecting outcome data, and changing practice on the basis of this data, should be routinely performed in rehabilitation clinics. This expectation is consistent with recommendations made by the United States Institute of Medicine in their 2012 report *Best Care at a Lower Cost*, which recommends that health care organizations standardize the administration of assessments (i.e. use the same assessments using standard methods) to improve care delivery, increase transparency of outcomes, strengthen public health, and generate new knowledge [32].

The EBPIS subscales provide information about the steps in EBP, however, they do not provide information about the required steps to implement EBP or the fidelity in which they are administered. Interestingly, item 1 and 14 are most closely related to implementation of EBP, but they clustered with knowledge exchange in the analysis. Future research on the EBPIS should explore the development of another subscale that reflects implementation activities, such as frequency of using evidence or practice guidelines to guide decisions, adaptation of guidelines, and collaboration with stakeholders to implement EBPs would provide a more comprehensive understanding of steps related to implementation.

### Use of evidence-based practices

In this study, we also identified rehabilitation clinicians’ self-reported use and perceptions of EBP within and between clinics in South Eastern Norway, and determined factors that may influence EBP. The EBPIS and its subscale scores indicate that common EBP activities among rehabilitation professionals in Norway are literature search, critical appraisal, and knowledge exchange. The findings in this study are similar to those of Melnyk and colleagues, who determined that nurses most commonly performed critical appraisal (31%) and had informal discussions about research (37%); however, they least commonly used evidence in practice (11%) [25]. Our findings were also similar to a Norwegian study of nurses which identified that the EBPIS average score was 7.8/72 [26]. In this study, Stokke and colleagues identified that the majority of EBP activities were performed in the areas of literature search, appraisal, and knowledge exchange and ~ 90% stated they did not systematically evaluate their own practice [26]. Our findings are consistent with these and other studies, indicating that conceptual use of research is more commonly reported than instrumental use (i.e use of research evidence in clinical practice) [25, 26, 33].

When assessing the factors associated with use of EBP, significant differences on the EBPIS and the Literature search/Appraisal and Knowledge Exchange subscales were noted between individuals with a Bachelors and Doctorate and a Master’s and Doctorate degree. No differences were noted between individuals with Bachelors and Master’s degrees or between any groups on the Practice Evaluation subscale. Other studies that examined whether differences in nurses’ use of EBP are associated with highest degree have conflicting results. One study found that those with an Associate’s degree scored lowest on the EBPIS and individuals with Doctorate degrees scored the highest [25], whereas another identified no difference between those with and without a higher level of education [26]. In a survey of PTs, differences in various aspects of literature search and critical appraisal were demonstrated between those with a baccalaureate as compared to a post-baccalaureate degree and between PTs with entry-level post-baccalaureate (i.e. first professional degree as a masters or doctorate) as compared to an advanced master’s or Doctorate (grouped together) [20]. None of these studies, however, assessed differences between clinicians with Bachelor’s and Master’s degrees. Research should examine differences in practice that result from obtaining advanced degrees (i.e PhD, EdD, or DHSc). Identifying key components of a curriculum or course that may facilitate increased EPB, including evaluation and change of current practice, is equally important. Professional programs, regardless of degree level, may benefit from adding education and training related to practice evaluation.

As another mechanism of professional development, clinicians often obtain a clinical specialty designation. These data indicate that individuals with specialty designation had significantly higher scores on the EBPIS as compared to those without one. Although significant, the actual difference in total scores (31.7 points vs. 30.4 points) was minimal. Further, no differences in subscale scores were identified. Specialty certification often includes demonstration of advanced knowledge and skills in rehabilitation, therefore, this finding should be investigated further.

Educational programs and organizations that employ rehabilitation providers in Norway should consider additional activities to support clinicians in increasing the instrumental use of EBP with patients. Programs that include multi-component interventions that combine strategies and target barriers to use of a new practice have demonstrated effectiveness [34]. An example of this type of program is the Physical therapist-drive Education for Action Knowledge translation (PEAK), which included acquiring leadership support and electronic resources, a 2-day EBP training workshop, small group work to adapt research to the local context and create a best practice list (5 months), review the best practice list, and agreement to implement it [35]. While the baseline mean total EBPIS score was 25.5 (slightly lower than the samples studied in our project), a statistically significant improvement in score was demonstrated immediately after the project conclusion with no changes at a 6 month follow-up [35]. The EBPIS scores statistically improved after the program, however, a chart audit indicated only one best practice behavior demonstrated a statistically significant change. The data also suggests additional support and knowledge translation processes (i.e. the Knowledge-to-Action framework), may be required to fully support implementation efforts [18, 36].

As previously discussed, EBPs should be administered with fidelity. Survey respondents indicated there may be variation in delivery of EBPs within each clinic and throughout the region, which indicates an opportunity to improve use of EBPs or the fidelity in which they are delivered may exist. Responses did not vary by hospital with the exception of a small and significant difference between participants at 1 hospital who reported a slightly lower agreement with use of the same outcome measures locally. By streamlining the use of EBPs within and between clinics, fidelity in delivery of EBP will be improved and unwarranted practice variation may be minimized. One mechanism to promote administration of EBPs with fidelity, is through use of a learning health care system (as previously described) which integrate clinical operations, research, and patient engagement, with a robust technology infrastructure [37]. This would facilitate monitoring of use of EBPs and facilitate robust data collection, analysis and rapid generation of practice-based evidence that has potential to improve the quality of rehabilitation. In order to build an infrastructure that supports the learning health care system, it is critical to systematically review literature, create clinical practice guidelines, adapt these guidelines for local application while defining key elements to ensure fidelity, and measure their use and impact.

Several limitations this study exist. First, we assessed the EBP perceptions of the participants. To identify actual use of EBP, a robust study design that observes practice is necessary. Additionally, other factors may contribute to EBP. Individual characteristics, such as knowledge, skills, and beliefs about of therapeutic interventions may impact adherence to practice recommendations [38]. Organizational factors such as leadership vision, style and communication may impact adoption [39]. Research also suggests that policy-makers, who encourage EBP through regulations, infrequently use published evidence to inform decisions [40]. More research is needed to better understand the contribution of each of these factors to EBP in rehabilitation. The Norwegian translation of the EBPIS has been used in a previous study of Norwegian practice, however, we are unaware of the translation methods used [26]. Additionally, 8 questions about EBPs were developed specifically for this survey, and were not previously validated. The survey was distributed to individuals who previously signed up for the email distribution list and the newsletter distributed by the Regional Center for Knowledge Translation in Rehabilitation, which may include a select group of individuals who are more interested in EBP than the general population. While social response bias may have also contributed to the outcome of the survey, respondents were aware that data would be de-identified. Given the relatively low score on the EBPIS, it is unlikely that this bias impacted the results. Lastly, various modes of survey distribution were used, therefore, we are unable to calculate an exact response rate.

## Conclusions

In summary, the primary EBP activities performed in rehabilitation are focused on literature search, critical appraisal, and practice evaluation. Individuals who have a Doctorate degree report significantly more EBP activities than those with a bachelors or master’s degree. Variability may exist in rehabilitation practice throughout Southeastern Norway. Research is needed to better understand the individual and organization contributions to EBP and the best methods to implement EBP across sites.

## Abbreviations

EBP: Evidence-based practice
EBPIS: Evidence-based practice implementation scale
KT: Knowledge translation
KTA: Knowledge-to-Action
OT: Occupational therapist
PT: Physical therapist
SLP: Speech language pathologist
